# Soluble P2X7 Receptor Plasma Levels in Obese Subjects before and after Weight Loss via Bariatric Surgery

**DOI:** 10.3390/ijms242316741

**Published:** 2023-11-25

**Authors:** Angelo Di Vincenzo, Marnie Granzotto, Andrea Graziani, Marika Crescenzi, Mirto Foletto, Luca Prevedello, Federico Capone, Roberto Vettor, Marco Rossato

**Affiliations:** 1 Clinica Medica 3, Department of Medicine—DIMED, University Hospital of Padova, 35128 Padua, Italy; angelo.divincenzo@unipd.it (A.D.V.); marnie.granzotto@unipd.it (M.G.); agraziani1995@gmail.com (A.G.); marikacrescenzi@hotmail.com (M.C.); caponefederico@hotmail.com (F.C.); roberto.vettor@unipd.it (R.V.); 2 Bariatric Surgery Unit, University Hospital of Padova, 35128 Padua, Italy; mirto.foletto@unipd.it (M.F.); luca.prevedello@aopd.veneto.it (L.P.)

**Keywords:** obesity, adipose tissue, P2X7 receptor, BMI, bariatric surgery, inflammation

## Abstract

Obesity is a systemic disease frequently associated with important complications such as type 2 diabetes and cardiovascular diseases. It has also been proven that obesity is a disease associated with chronic low-grade systemic inflammation and that weight loss improves this low-grade chronic inflammatory condition. The P2X7 purinergic receptor (P2X7R), belonging to the family of the receptors for extracellular ATP, is a main player in inflammation, activating inflammasome and pro-inflammatory cytokine production. In this study, we evaluated the plasma levels of soluble P2X7R (sP2X7R) measured in a group of obese patients before and one year after bariatric surgery. Furthermore, we evaluated the relation of sP2X7R to inflammatory marker plasma levels. We enrolled 15 obese patients who underwent laparoscopic sleeve gastrectomy, evaluating anthropometric parameters (weight, height, BMI and waist circumference) before and after surgery. Moreover, we measured the plasma levels of inflammatory markers (CRP, TNFα and IL-6) before and after weight loss via bariatric surgery. The results of our study show that one year after bariatric surgery, obese patients significantly decrease body weight with a significant decrease in CRP, TNF-alfa and IL-6 plasma levels. Similarly, after weight loss, obese subjects showed a significant reduction in sP2X7R plasma levels. Moreover, before surgery, plasma levels of sP2X7R were inversely related with those of CRP, TNF-alfa and IL-6. Given the role of P2X7R in inflammation, we hypothesized that, in obese subjects, sP2X7R could represent a possible marker of chronic low-grade inflammation, hypothesizing a possible role as a mediator of obesity complications.

## 1. Introduction

Obesity and its related complications have reached epidemic diffusion, and its prevalence is every increasing worldwide [1]. It is well known that obesity is characterized by a state of low-grade chronic inflammation that plays an important role in the pathogenesis of obesity-related systemic complications such as cardiovascular diseases, hypertension, diabetes mellitus and some forms of cancer [2]. In the obese subject, several inflammatory cytokines are released by inflammatory cells hosted within the adipose tissue but also directly by adipocytes themselves [3,4]. These inflammatory cytokines exert direct and indirect effects on the body’s metabolic processes, altering glucose uptake in insulin-sensitive tissues [5] and leading to the typical metabolic complications of obesity such as diabetes mellitus [1].

In humans, the expression of many different inflammatory cytokines is positively correlated with body mass index (BMI) and body fat mass [6]. In recent years, the role of the P2X7 purinergic receptor (P2X7R) in the initiation of the inflammatory process has become increasingly important [7,8,9,10]. P2X7R is a member of the P2X family of purinergic receptors expressed in many different cell types and, in particular, in the cells of the immune system, with a consolidated role in the modulation of inflammation [9].

Adipose tissue expresses the P2X7R that is functionally activated by adenosine-tris-phospate (ATP) and benzoyl-ATP (BzATP), inducing the secretion of different inflammatory cytokines such as tumor necrosis factor alpha (TNFα) and interleukin-6 (IL-6) [11,12,13] and participating in the impairment of insulin signaling [14]. Furthermore, P2X7R activation by BzATP activates lipolysis [13], although contrasting effects have been reported [15].

ATP is generally known as a pro-inflammatory molecule, acting mainly via the activation of P2X7R on target cells after its release by damaged cells or via active release mechanisms [16]. Thus, it is possible that P2X7R might affect different functions in adipocytes, and these alterations could be amplified during pathological conditions characterized by systemic adipose tissue expansion such as obesity.

The chronic low-grade systemic inflammation present in obesity due to the release of inflammatory cytokines seems to play an important role in the pathogenesis of obesity-associated systemic and metabolic complications [17,18,19]. Recently, it has been reported that P2X7R is released and detectable in circulation, correlating with C-reactive protein (CRP) plasma levels in patients with different inflammatory conditions [20].

In the present study, we have determined the shed P2X7R (sP2X7R) plasma levels in a group of obese patients before and after body weight reduction due to laparoscopic sleeve gastrectomy, evaluating possible correlations with common systemic inflammatory markers.

## 2. Results

Table 1 reports the anthropometric characteristics of the studied patients, with basal elevated body weight, BMI and waist circumference together with basal plasma levels of CRP, IL-6 and TNFα that were elevated before bariatric surgery.

As expected, after sleeve gastrectomy, we observed a rapid and progressive reduction in body weight (Table 1) together with a significant reduction in the plasma levels of the inflammatory markers (CRP, IL-6 and TNFα) (Table 1).

**Figure 1:**
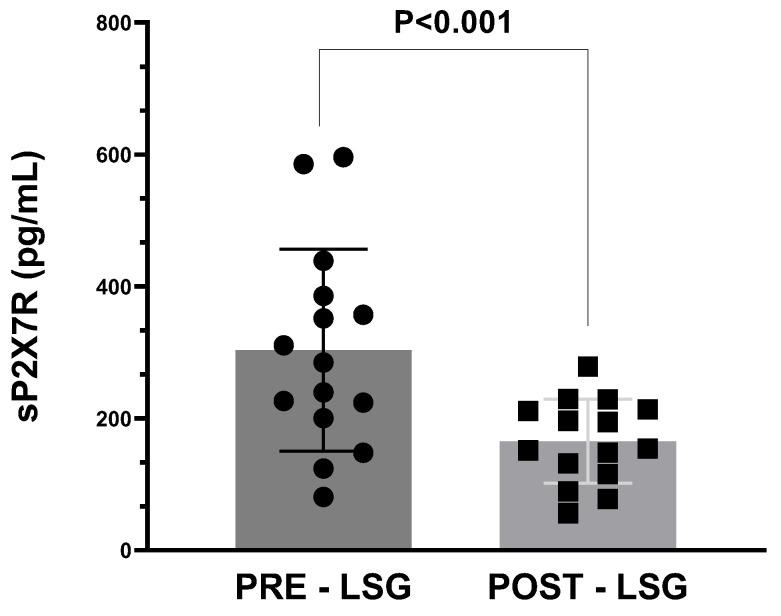
Plasma levels of sP2X7R in obese subjects before (PRE) and after (POST) body weight reduction via laparoscopic sleeve gastrectomy (LSG). Data analysis was performed with Wilcoxon matched pairs test. A *p*-value < 0.05 was considered as statistically significant.

Similarly, sP2X7R plasma levels were also significantly reduced in obese subjects after weight loss via bariatric surgery (*p* < 0.001, Figure 1).

**Figure 2:**
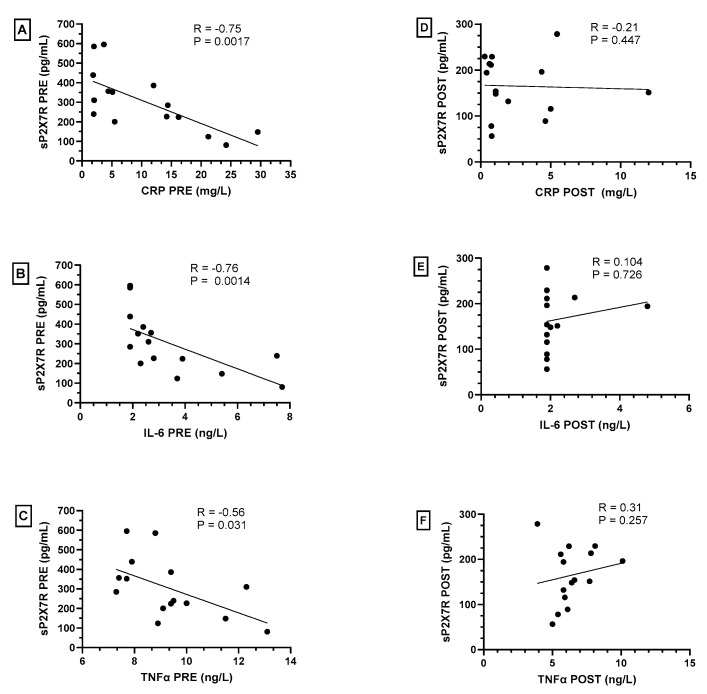
Spearman’s correlations between plasma levels of shed P2X7 receptor (sP2X7R) with C-reactive protein (CRP), IL-6 and TNFα in obese subjects before (PRE—(**A**–**C**)) and after (POST—(**D**–**F**)) bariatric surgery. A *p*-value < 0.05 was considered statistically significant.

Interestingly, we observed a significant inverse correlation between plasma levels of sP2X7R and those of CRP, IL-6 and TNFα in obese subjects before weight loss via bariatric surgery (Figure 2A–C, respectively). On the contrary, after bariatric surgery and significant weight loss, we did not observe any statistically significant correlation between these parameters (Figure 2D–F, respectively).

**Figure 3:**
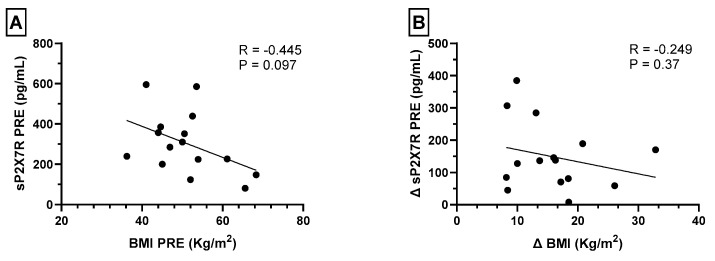
(**A**) Spearman’s correlations between plasma levels of shed P2X7 receptor (sP2X7R) and BMI before bariatric surgery. (**B**) Spearman’s correlations between the difference in sP2X7R and BMI values before and after surgery (∆sP2X7R and ∆BMI, respectively). A *p* value < 0.05 was considered statistically significant.

In order to determine if the decrease in sP2X7R plasma levels after bariatric surgery was related to weight loss, we analyzed the relation between sP2X7R plasma levels and BMI before bariatric surgery, without observing any significant correlation (Figure 3A). Furthermore, we performed a correlation analysis between the difference in sP2X7R and BMI values before and after surgery (∆sP2X7R and ∆BMI, respectively) without observing any significant relation (Figure 3B).

## 3. Discussion

The worldwide incidence of obesity is ever increasing, together with its associated systemic complications, such as type 2 diabetes mellitus, cardiovascular diseases, dyslipidemias and some cancers, thus representing a huge threat for people’s health [21].

Obesity is considered a systemic disease associated with low-grade chronic inflammation [22], and a huge wealth of studies have demonstrated the primary role of chronic inflammation in the pathophysiological mechanisms leading to the development of chronic complications of obesity [23].

The main culprits of this low-grade chronic inflammatory state of obesity are abundant adipose tissue, through the production of pro-inflammatory adipokines, and immune cells, primarily macrophages and lymphocytes, which are resident in or migrate within the adipose tissue attracted by chemotactic stimuli directly secreted by the adipocytes [24].

To confirm the close association of obesity with inflammation, it is well known that obese subjects almost always show moderately elevated plasma levels of inflammatory markers such as CRP and IL-6 [25]. Furthermore, the loss of fat mass, induced by lifestyle modifications, pharmacological therapy and/or bariatric surgical treatment, determines a reduction in inflammatory cytokine levels and an improvement in the inflammatory state that characterizes obesity, confirming the primary role of adipose tissue in the pathogenesis of this condition [26].

It has been demonstrated that the purinergic receptor P2X7R plays a primary role in many different inflammatory processes underlying numerous pathologies (ocular diseases, systemic autoimmune diseases, liver diseases, COVID-19-related ARDS) through activating many different signaling pathways, ultimately determining the activation of inflammatory cells and/or the direct synthesis of pro-inflammatory cytokines [8,9,27].

P2X7R, a plasma membrane receptor activated by extracellular ATP, derives from the assembly of three identical subunits that can be shed via proteolytic cleavage [28] or associated with plasma membrane-derived microvesicles and microparticles in different cell types [29]. Alternatively, it is possible that the full trimeric P2X7R is shed from cells as for the single subunits, as recently demonstrated by Giuliani et al. [20]. In their study, Giuliani et al. showed that, in addition to its expression in many different tissues, P2X7R is released within the bloodstream and is associated with microvesicles and/or microparticles of cellular origin [20]. The same study demonstrated the presence of a direct linear relationship between serum concentrations of sP2X7R and CRP in specific inflammatory conditions [20]. Recently, we have shown that human adipocytes express P2X7R and that extracellular ATP induces the production and secretion of IL-6 [4]. On the other hand, it has been previously shown that P2X7R is expressed also in CD4+ T-cells deriving from peripheral blood and adipose tissue, with increased expression in subjects with an elevated BMI [30].

In the present study, using a recently commercialized ELISA kit directed to the full molecule of sP2X7R, we have shown that in subjects with obesity, weight loss after bariatric surgery induced a significant decrease in blood sP2X7R concentrations compared to the pre-intervention concentrations.

We have also analyzed the relationships between plasma concentrations of sP2X7R and the most common plasmatic inflammatory markers associated with the chronic low-grade inflammation present in obesity, such as CRP, TNFα and IL-6. All these inflammatory parameters were significantly decreased after weight loss, as for sP2X7R levels. Nonetheless, serum concentrations of sP2X7R were inversely correlated with those of CRP, TNFα and IL-6.

The inverse relationships between the serum concentrations of sP2X7R and those of CRP, TNFα and IL-6 are currently unexplained but could be linked to the small number of samples or to a specific effect of the particular inflammatory state that characterizes obesity, with different effects on the production of each specific inflammatory marker. To this regard, there are few published studies regarding the shedding of P2X7R within the plasma in humans [4,31]. In their elegant study, Giuliani et al. compared sP2X7R plasma levels in patients suffering from diseases characterized by different inflammatory states, such as infectious disease, brain/heart ischemia and cancer. Interestingly, the authors observed a positive linear correlation between serum sP2X7R and CRP, a serum marker of acute inflammation, in patients with acute inflammatory conditions such as infection/sepsis. On the contrary, the authors did not observe any relationship between sP2X7R and CRP plasma levels in patients with cancer, a condition generally characterized by low-grade chronic inflammation. Similarly, Garcia-Villalba et al. observed a direct correlation between sP2X7R and CRP plasma levels during the acute phase in COVID-19 patients [31].

The quite interesting observation of our study is represented by the fact that after significant weight reduction induced via bariatric surgery, obese patients showed a significant reduction in sP2X7R plasma levels, along with a reduction in well-known markers of systemic inflammation such as CRP and TNFα, as usually expected after weight loss, to signify a reduction in the (still unknown) inflammatory stimuli produced in obesity.

The pathophysiological significance of the elevation of serum sP2X7R in a low-grade chronic inflammatory state such as obesity is unclear. To this regard, it is known that plasma membrane receptors for cytokines can be shed into circulation through proteolytic mechanisms or released in association with plasma membrane-derived microvesicles or microparticles influencing the signaling by the cognate agonist [32,33,34]. Furthermore, the measurement of soluble cytokine receptors is gaining interest for the differential diagnosis of selected immune-mediated diseases [20].

It is not possible at the moment to establish which cells release sP2X7R within circulation. Previous studies have demonstrated that dendritic cells are able to release sP2X7R after stimulation with ATP [29]. ATP is present in the peri-cellular space in tissues affected by inflammation under conditions allowing for the activation of P2X7R, and, thus, there are conditions allowing for the release of microvesicles containing P2X7R itself or its subunits [13]. It is highly probable that, in addition to dendritic cells, many other cell types, mainly immune cells, are able to shed P2X7R within the plasma. Human adipocytes, which express P2X7R and functionally respond to stimulation with extracellular ATP [4], could be able to release this receptor within circulation [13]. The lack of any significant relation between the degree of the reduction in sP2X7R plasma levels (ΔsP2X7R) and BMI (ΔBMI) induced via bariatric surgery seem to exclude a role for adipose tissue as the main determinant of sP2X7R release, although the reduced number of patients considered in the present study could have hampered this result. Nonetheless, the observation that obese subjects show a significant decrease in serum sP2X7R after important weight loss could suggest the use of plasma sP2X7R concentration as a non-specific inflammatory marker in obesity that can be monitored during the clinical management of this disease, possibly being a diagnostic and/or a prognostic marker of this disease.

Finally, the increased release of sP2X7R in obese subjects, regardless of the tissue responsible for the release, could transfer P2X7R to other cells distant from the site of secretion. Considering the role of P2X7R in inflammation, the shedding of P2X7R within the circulation might represent a potential “tranferrable” pro-inflammatory stimulus.

Further studies will be necessary to clarify the cell(s) responsible for sP2X7R release in subjects with obesity and if this “transferrable” sP2X7R has a role in obesity and, in particular, in the pathogenesis of the well-known important systemic complications of this disease.

## 4. Materials and Methods

### 4.1. Patients

We retrospectively analyzed data from fifteen obese patients (6 males and 9 females), with a mean age of 46.1 ± 7.6 years, previously treated with laparoscopic sleeve gastrectomy (LSG). All subjects had their clinical history recorded and underwent and a physical examination, routine blood tests, the measurement of body weight, the measurement of BMI and abdominal circumference and an evaluation of biochemical status. Three weeks before surgery, all patients were put on a balanced very-low-calorie diet (800 kcal/day). Following surgery, all patients were regularly scheduled for post-bariatric follow-up with clinical and biochemical assessments after 1, 3, 6 and 12 months, to monitor any potential complications and, in particular, nutritional deficiencies. In the early stages of recovery after surgery, a liquid diet was recommended before gradually incorporating semi-liquid foods approximately one month after surgery before the transition to a balanced diet. Additionally, all patients were prescribed multivitamins and mineral supplements for the post-surgical period. LSG was performed by the same surgical team in adult obese patients (>18 and <60 years old), with a BMI greater than 35 kg/m^2^ in the presence of complications or with a BMI greater than 40 kg/m^2^ with or without complications, according with the NIH consensus criteria for bariatric surgery [35]. Further exclusion criteria regarded patients with previous bariatric or gastric surgery and active gastric ulcer disease. Absolute exclusion criteria included alcohol addiction and severe psychiatric disorders. Clinical and anthropometrical parameters and plasma levels of sP2X7R, CRP, IL-6 and TNFα in obese patients were evaluated before and one year after bariatric surgery.

### 4.2. Blood Sample Collection

Fasting venous blood samples were obtained in all participants pre-operatively in the morning between 8 a.m. and 9 a.m., after 8 h fasting. Plasma collected in K3-EDTA tubes was separated after centrifuging at 3000 rpm for 15 min and then transferred and stored in sterile Eppendorf tubes at −80 °C for the subsequent analyses. Sample collection and processing were performed in a similar manner in each obese subject at a one-year follow-up visit after LSG.

### 4.3. Plasma Measurements

The plasma concentration of sP2X7R was determined utilizing the P2X7R ELISA kit (CUSABIO, Houston, TX, USA), following manufacturer’s instructions. Optical density was measured spectrophotometrically at 450 nm.

Tumor necrosis factor alpha (TNFα), interleukin-6 (IL-6) and hsCRP plasma levels were determined at the Central Laboratory of the University Hospital of Padova using commercial kits. Each determination was run in duplicate.

### 4.4. Statistical Analysis

Mean values with standard deviations (SD) were calculated to describe the data obtained before and after weight loss. All the variables were tested for normality with a graphic method (histograms) using Jamovi (version 2.3.21). All continuous variables were analyzed using a Wilcoxon or paired *t*-test according to their distribution. Simple linear regression analysis and Spearman’s correlation coefficients were calculated for the association between sP2X7R and CRP, IL-6 and TNFα plasma levels before and after weight reduction via bariatric surgery. A *p* value < 0.05 was considered statistically significant. The statistical analysis was carried out using GraphPad PRISM software (version 9.5.1. GraphPad Software Inc., La Jolla, CA, USA).

## 5. Conclusions

In this study we demonstrated that obese subjects present detectable plasma levels of sP2X7R that are significantly reduced after weight loss via bariatric surgery. The possible role of the release of sP2X7R within the plasma in the pathogenesis of the different complications of obesity requires further experimental and clinical studies.

## Figures and Tables

**Table 1:** Body weight, body mass index (BMI), waist circumference (WC), plasma levels of C-reactive protein (CRP), tumor necrosis factor alpha (TNFα) and interleukin-6 (IL-6), measured before (PRE-LSG) and after body weight reduction via sleeve gastrectomy s(POST-LSG). Data have been reported as medians. Interquartile range values have been reported in brackets. * Data analyzed with paired *t*-test; ^#^ Data analyzed with Wilcoxon matched pairs test. A *p*-value < 0.05 was considered statistically significant.

	PRE-LSG	POST-LSG	*p*
Body weight (kg)	138 (37.4)	102 (31)	<0.00001 *
BMI (kg/mq)	50.5 (9.3)	34 (8.3)	<0.00001 *
WC (cm)	138 (23)	107 (19)	<0.00001 *
CRP (mg/L)	5.5 (14.2)	1.04 (4.1)	<0.001 ^#^
TNFα (ng/L)	9.1 (2.3)	6.1 (2.1)	<0.005 ^#^
IL-6 (ng/L)	2.6 (2)	1.9 (0.1)	<0.005 ^#^

## Data Availability

The data presented in this study are available on request from the corresponding author. The data are not publicly available due to reasons of confidentiality.
